# Effect of Anode Interfacial Modification by Self-Assembled Monolayers on the Organic Solar Cell Performance

**DOI:** 10.1021/acsomega.3c04081

**Published:** 2024-02-07

**Authors:** Adem Mutlu, M. Zeliha Arkan, Mustafa Can, Cem Tozlu

**Affiliations:** † Solar Energy Institute, Ege University, Izmir 35100, Turkey; ‡ Institute of Chemistry, University of Silesia in Katowice, Szkolna 9, Katowice 40-006, Poland; § Graphene Application and Research Center, Izmir Katip Celebi University, Cigli, Izmir 35620, Turkey

## Abstract

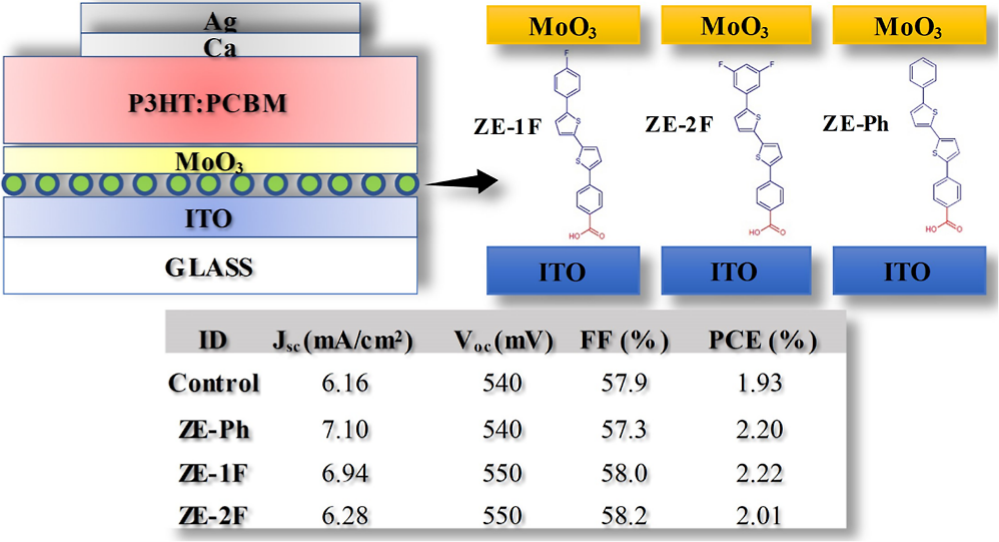

A series of self-assembled monolayer (SAM)-based benzoic acid derivatives such as 4-[5′-phenyl-2,2′-bitien-5-yl] benzoic acid (ZE-Ph), 4-[5′-(4-fluorophenyl)-2,2′-bitien-5-yl]benzoic acid (ZE-1F), and 4-[5′-(3,5-difluorophenyl)-2,2′-bitien-5-yl]benzoic acid (ZE-2F) were synthesized to use an interlayer between an ITO electrode and a MoO_3_ thin film layer in an organic solar cell (OSC) having poly-3 hexylthiophene (P3HT): [6,6]-phenyl C_61_ butyric acid methyl ester (PC_61_BM) blend. The work function and surface wetting properties of the ITO were tuned by SAM molecules. The power conversion efficiency of fabricated OSC devices was improved compared to that of the control device from 1.93 to 2.20% and 2.22% with ZE-Ph and ZE-1F-modified ITO electrodes, respectively. The short-circuit current density (*J*_sc_) was increased from 6.16 to 7.10 mA/cm^2^ and 6.94 mA/cm^2^ with control, ZE-Ph, and ZE-1F-modified solar cells, respectively. The increase in short-circuit current density (*J*_sc_) shows that the hole-transporting properties between ITO and MoO_3_ were improved by the use of ZE-Ph and ZE-1F compared with that of the ITO/MoO_3_ electrode configuration. The open-circuit voltage (*V*_oc_) of the SAM-modified ITO-based devices was also improved compared with the *V*_oc_ of unmodified ITO-based devices. These results show that using a monolayer as an interlayer in OSCs is an important strategy to improve the performance of OSCs. All the device parameters were characterized by Kelvin probe force microscopy, cyclic voltammetry, contact angle, and *I*–*V* measurements.

## 1 Introduction

Conjugated-polymer organic semiconductor-based organic solar cells (OSCs) are one of the most promising technologies to be alternative energy sources because of their versatility in production methods, low manufacturing costs, and applicability on flexible surfaces.^1−6^ One of the most effective OSC device structures is bulk heterojunction (BHJ) solar cells composed of a percolating network of photoactive donor and acceptor organic compounds between transparent conductive and metallic electrodes. Tremendous strategies such as the synthesis of novel light-harvesting organic materials, the improvement of surface morphology, and interface engineering have been developed to improve the solar cell efficiency.^7−11^ The interface engineering also enhances photoinduced charge collections selectively with the appropriate buffer layers without recombination due to an adjustment in energy level between the photoactive layer and buffer layers. The energy levels of the donor material and acceptor material must exhibit ohmic behavior with metal electrodes to remove charge carriers from the OSC without recombination.^12−14^ A number of methods for the alignment of the interfacial properties have been applied to increase the current density in OSCs. One of them is to use buffer layers such as conductive polymers, metal oxides, small molecules, etc.^15^ These buffer layers are used as either hole-blocking layers or electron-blocking layers due to matching the energy level of buffer layers with the lowest unoccupied molecular orbital or the highest occupied molecular orbital (HOMO) level of the organic semiconductors.^16^

Conductive indium tin oxide (ITO) electrodes are widely used in OSCs and various applications due to their extreme properties such as transparency in the visible region, ease of patterning, and low electrical resistivity. The ITO layer is commonly used as an anode electrode in conventional OSC geometry. However, the rough surface and high work function value of ITO are the main disadvantages of the OSC applications.^17,18^ To overcome ITO’s disadvantages, the deposition of a buffer transport layer on ITO, such as polymeric materials or metal oxides, is one of the most common methods to improve OSC’s performance.^19−23^ A conductive polymer poly(3,4-ethylenedioxylene thiophene)/poly(styrene sulfonic acid) (PEDOT:PSS) is widely used as an anode buffer layer on ITO in conventional OSC construction. However, the acidity and hygroscopicity of PEDOT:PSS lead to deterioration in the long-term performance of the devices.^24^ MoO_3_ is one of the most remarkable materials among the metal oxides and an alternative buffer layer to PEDOT:PSS because of its chemical stability, nonacidic formation, and easy thin film formation from both the sol–gel and evaporation processes. Long-term performances of OSCs were improved by the use of a MoO_3_ anode buffer layer compared to that of PEDOT:PSS.^25,26^

An atomic surface modification of the charge transport layer with self-assembled monolayers (SAMs) is a cost-effective technique to modify the interface in optoelectronic devices. The SAMs have been studied extensively in recent years due to their excellent chemical and physical properties on the surface applications such as surface protection, biosensing, and hybrid solar cells. It is well-known that the threshold voltage and the current density have been developed in many organic electronic devices such as organic light-emitting diodes and OSCs by the modification of the ITO layer with SAMs.^27−29^ Modifying the surface of the ITO with a SAM is a very effective method to effectively collect more charge carriers by making the rough surface of the ITO smoother. Modification of the ITO surface results in a decrease of the recombination and an increase of *J*_sc_.^30−33^ Although the metal oxide/active layer interface is significant for device efficiency, the ITO/metal oxide interface is also crucial.^34^ According to our knowledge up to now, the modification of the ITO/MoO_3_ interface by SAMs has not been reported in the literature on the OSC application. Furthermore, considering that metal oxide materials tend to form aggregates on the surface during coating, it is essential to address and solve this issue.^35^ Therefore, the problem of defects at the ITO/MoO_3_ interface is worth solving, and using methods such as SAM to achieve a better passivation quality is an effective, easy, and cheap method to further improve the device’s performance. The SAM molecules used as interlayers between ITO/metal oxide interfaces are rarely reported.

In this work, we have successfully exhibited the improvement in solar cell efficiency through the surface modification of ITO using a range of boronic acid derivatives containing fluoro terminal groups. These derivatives served as an anode interlayer to facilitate an efficient hole injection. To the best of our knowledge, the SAM/MoO_3_ hybrid hole-selective electrode studies in BHJ OSC have not been reported in the literature yet. Thus, novel SAM structures were synthesized with benzoic acid derivatives such as 4-[5′-phenyl-2,2′-bitien-5-yl] benzoic acid (ZE-Ph), 4-[5′-(4-fluorophenyl)-2,2′-bitien-5-yl]benzoic acid (ZE-1F), and 4-[5′-(3,5-difluorophenyl)-2,2′-bitien-5-yl]benzoic acid (ZE-2F) and inserted between ITO and MoO_3_ layers.

## 2 Experimental Methods

### 2.1 Materials

5-Bromo-5′-(4,4,5,5-tetramethyl-3,2-dioxaborolan-2-yl)-2,2′-bithiophene was purchased from TCI. [4-(Methoxycarbonyl) phenyl] boronic acid, benzene boronic acid, 4-fluorobenzene boronic acid, 3,5-difluorobenzene boronic acid, 3,4,5-trifluorobenzene boronic acid, *N*,*N*-dimethylformamide (DMF), and 1,2-dimethoxy ethane (DME) were acquired from Alfa-Aesar. [1,1′-Bi(diphenylphosphino) ferrocene]dichloropalladium(II) and MoO_3_ were purchased from Sigma-Aldrich. Potassium carbonate was purchased from Riedel de Haen. Poly-3 hexylthiophene (P3HT) and PC_61_BM were acquired from Luminescence Technology Company (LUMTEC).

### 2.2 Synthesis

**Scheme 1:**
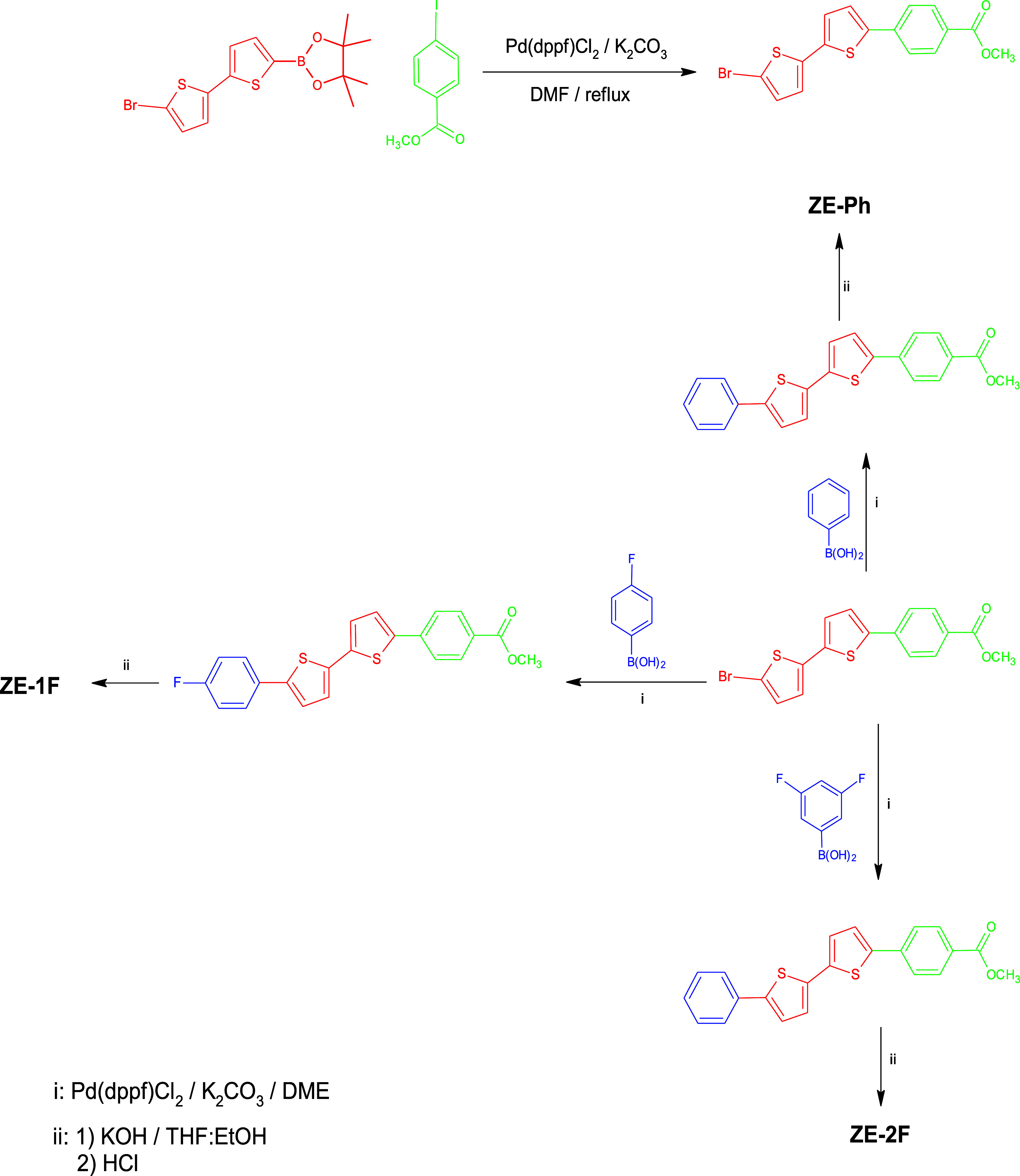
Synthetic Route to ZE-Ph, ZE-1F, and ZE-2F SAM Molecules

All commercial materials were utilized without undergoing any purification. Prior to use, all glassware was dried in an oven, and all reactions were conducted under an inert (N2) atmosphere. The synthetic route of SAM molecules is given in Scheme 1.

### 2.3 Synthesis of Methyl 4-(5′-Bromo-2,2′-bit-5-yl) Benzoate

A solution was prepared by dissolving 5-bromo-5′-(4,4,5,5-tetramethyl-3,2-dioxaborolan-2-yl) −2,2′-bithiophene (200 mg, 0.54 mmol) and methyl 4-bromo benzoate (135 mg, 0.75 mmol) in 20 mL of DMF inside a Schlenk flask, followed by the addition of K_2_CO_3_ (2 mL, 1 M) under a nitrogen (N_2_) atmosphere. The final mixture was heated to 90 °C and stirred overnight. The reaction terminated with the control of TLC analysis and was extracted with water and dichloromethane. The solvent was evaporated, and the resulting product was subjected to purification through column chromatography (SiO_2_, CH_2_Cl_2_/*n*-hexane:1/1) to afford yellow powder as a product.

### 2.4 Synthesis of Methyl 4-(5′-Phenyl[2,2′-bithiophene]-5-yl) Benzoate (ZE-Ph)

A solution was prepared in a Schlenk flask by dissolving a mixture of 4-(5′-bromo-2,2′-bit-5-yl) benzoate (200 mg; 0.53 mmol) and benzene boronic acid (96.3 mg; 0.79 mmol) in 20 mL of DME. When the temperature increased to 90 °C, Pd(dppf)Cl_2_ and K_2_CO_3_ (2 mL; 1 M) were added to the mixture and stirred to subject it to the Suzuki coupling reaction. The reaction was terminated with the control of TLC, and the final product was extracted with CH_2_Cl_2_ and water. The organic solvent was eliminated by using a rotary evaporator, and the resulting crude product was subjected to purification via column chromatography (using SiO_2_ and a CH_2_Cl_2_/*n*-hexane mixture in a ratio of 1:1) to yield a yellow powder. Subsequently, the product was dissolved in a THF–ethanol mixture (1:1, V/V), followed by the addition of KOH (0.5 mL, 1 M) to the solution. After an overnight reflux reaction, the solvents were evaporated, and the pH of the mixture was adjusted to a range of 3–4 using 10 mL of pure water and 1 M HCl. Finally, the resulting precipitated organic yellow product was filtered, washed with pure water, and left to dry overnight.

### 2.5 Synthesis of 4-[5′-(4-Fluorophenyl)-2,2-bithiophene-5-yl] Benzoic Acid (ZE-1F)

In a Schlenk flask, a solution was prepared by dissolving 200 mg (0.53 mmol) of 4-(5′-bromo-2,2′-bit-5-yl) benzoate and 110.5 mg (0.79 mmol) of 4-fluorobenzene boronic acid in 20 mL of DME. When the temperature increased to 90 °C, Pd(dppf)Cl_2_ (22 mg; 0.03 mmol) and K_2_CO_3_ (1.0 mL; 1 M) were added to the mixture and stirred to subject it to the Suzuki coupling reaction. The reaction was terminated with the control of TLC, and the final product was extracted with CH_2_Cl_2_ and water. The crude product obtained after the removal of the organic solvent using a rotary evaporator was subjected to purification through column chromatography using SiO_2_ with a mixture of CH_2_Cl_2_ and *n*-hexane in a ratio of 1:1. This process resulted in the formation of a yellow powder as the purified product. Subsequently, the product was dissolved in a mixture of THF and ethanol in a 1:1 volume ratio, and then, a KOH solution with a concentration of 0.5 M was added to the solution. The mixture was refluxed overnight, and the solvents were evaporated. To adjust the pH of the mixture to a range of 3–4, 10 mL of pure water and 1 M HCl were used. Finally, the organic yellow product that precipitated was filtered, washed with pure water, and left to dry overnight.

### 2.6 Synthesis of 4-[5′-(4-Fluorophenyl)-2,2′-bithiophene-5-yl] Benzoic Acid (ZE-2F)

In a Schlenk flask, 200 mg (0.53 mmol) of 4-(5′-bromo-2,2′-bithien-5-yl) benzoate and 109 mg (0.69 mmol) of 3,5-difluorobenzene boronic acid were dissolved in 20 mL of DME. When the temperature increased to 90 °C, Pd(dppf)Cl_2_ (22 mg; 0,03 mmol) and K_2_CO_3_ (1 mL; 1 M) were added to the mixture and stirred to subject it to the Suzuki coupling reaction. The reaction was terminated with the control of TLC, and the final product was extracted with CH_2_Cl_2_ and water. The crude product obtained after the removal of the organic solvent using a rotary evaporator was subjected to purification through column chromatography using SiO_2_ and a mixture of CH_2_Cl_2_ and *n*-hexane in a ratio of 1:1. This process resulted in the isolation of a yellow powder as the final product. Subsequently, the product was dissolved in a mixture of THF and ethanol in equal volumes (1:1, V/V), and KOH solution (0.5 M; 1 M) was added to the solution. The reaction mixture was refluxed overnight, followed by the evaporation of solvents. The pH of the mixture was adjusted to a range of 3–4 using 10 mL of pure water and 1 M HCl. Finally, the organic yellow product that precipitated was filtered, washed with pure water, and left to dry overnight.

### 2.7 Fabrication of the Device

**Figure 1:**
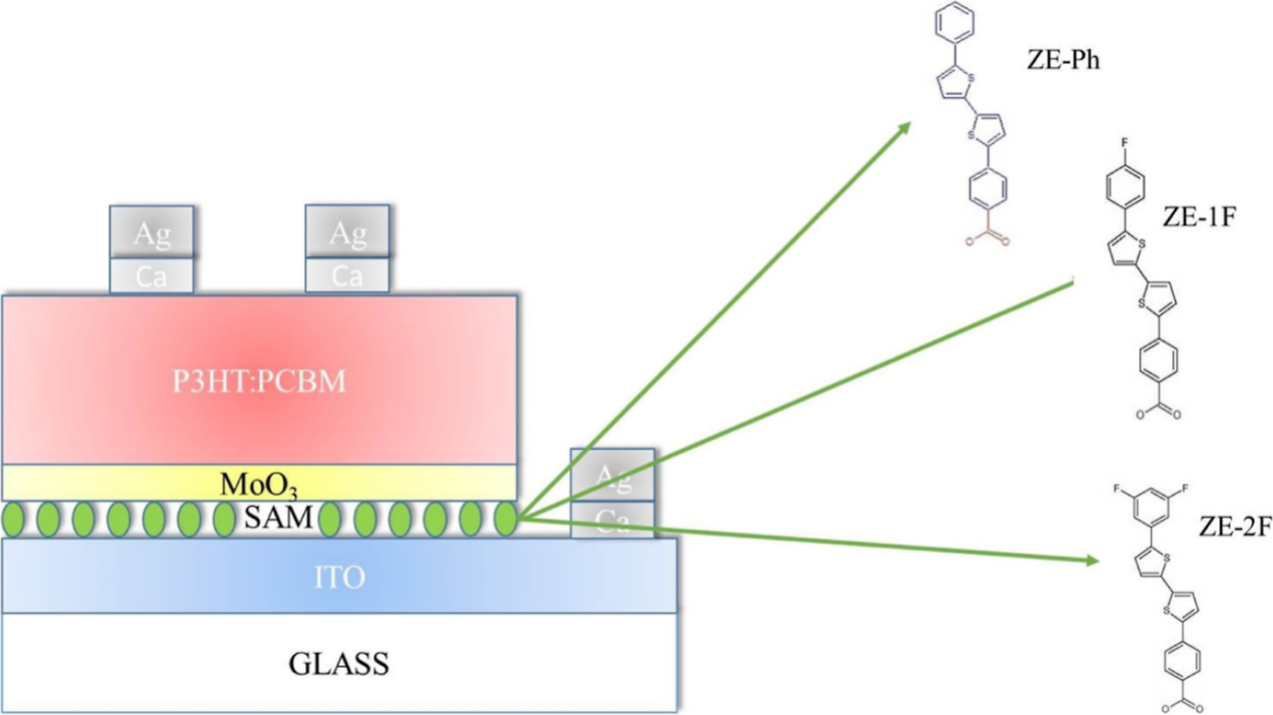
Schematic illustration of the OSC structure with the SAM-modified ITO anode layer and the structure of the SAM molecules.

Figure 1 shows a schematic representation of the fabricated OSC devices with ITO/SAM/MoO_3_ and ITO/MoO_3_ configurations for the anode electrode. The ITO substrates, with a sheet resistance of 15 Ω/sq, underwent a thorough cleaning process in an ultrasonic bath using pure water, followed by acetone and isopropyl alcohol rinses. Subsequently, a N_2_ flow gas was employed to dry ITO substrates. Then, UV ozone treatment was applied to the ITO substrates for 30 min. To deposit the SAMs on the ITO surface, the ITO substrates were immersed in 1 mM solutions (in DMF) of ZE-Ph, ZE-1F, and ZE-2F for 24 h at room temperature. After SAM formation, both the bare and SAM-modified ITO substrates were transferred into a glovebox. A 10 nm-thick buffer layer of the MoO_3_ film was thermally evaporated onto the ITO/SAM and bare ITO substrates at a deposition rate of 0.2 Å/s under a high vacuum of 7 × 10^–7^ Torr. For the active layer, a blend of PCBM/P3HT (1:0.8 w/w) was spin-coated at a spin rate of 800 rpm onto the ITO/MoO_3_ and SAM-modified ITO/SAM/MoO_3_ anode layers. To complete the devices, 20 nm of calcium (Ca) and 100 nm of silver (Ag) were thermally evaporated as top electrodes using a shadow mask under a high vacuum of 7 × 10^–7^ Torr.

### 2.8 Measurements

The CHI 660 E potentiostat was used for conducting cyclic voltammetry (CV) measurements. NTEGRA Solaris was used for performing Kelvin probe force microscopy (KPFM) characterizations. Contact angle measurements were carried out using water droplets and a Data Physics OCA 50 instrument. The dark and illumination *J*–*V* electrical characterizations of the OSCs took place inside the glovebox. The current density and voltage values of OSCs were obtained by using a Keithley 2400 source meter under dark and illumination conditions. The OAI Trisol (class AAA, 1000W), combined with an AM1.5 filter, was used as the light source, and a light intensity of 1000 W/m^2^ was maintained in all measurements. The light intensity was calibrated by using a certified Si reference solar cell before measurements to obtain the *J*–*V* plots. The contact potential differences (CPDs) were measured using a KPFM technique with the Ntegra Solaris instrument from NTD-MDT. At room temperature, the CPDs were measured relative to the conductive Pt (NSG03/Pt) electrode, employing a force constant ranging from 0.35 to 6.06 N/m and a resonance frequency spanning from 47–150 kHz. To conduct incident photon-to-current efficiency (IPCE) analysis, an Enlitech QE-R system equipped with a 75 W xenon arc lamp source was used.

## 3 Results and Discussion

**Figure 2:**
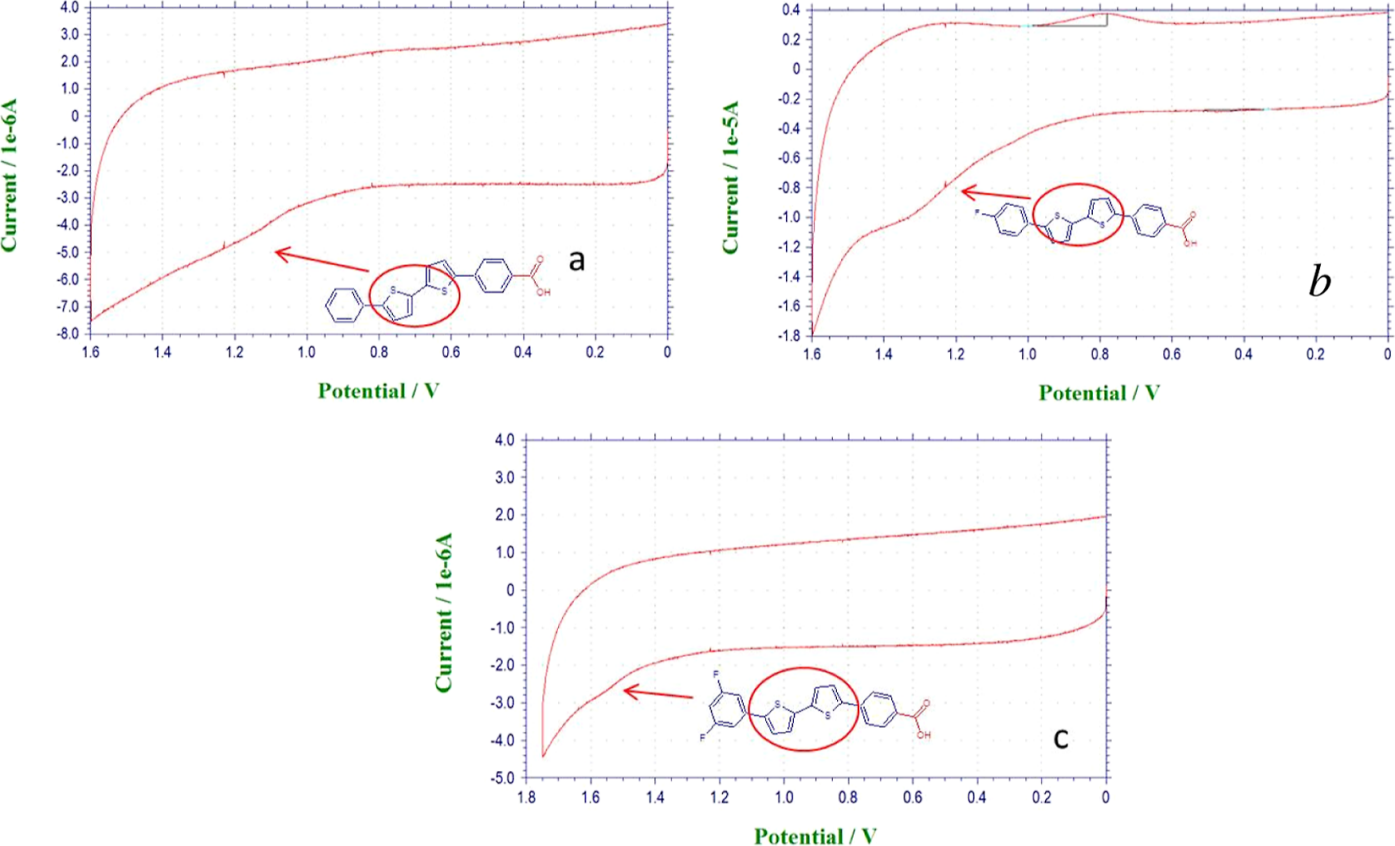
Voltammetric curves of (a) ZE-Ph, (b) ZE-1F, and (c) ZE-2F molecules on ITO electrode surfaces.

To analyze the SAM modification of ITO, the oxidation potential shift of ITO/SAM electrodes was examined by CV measurements, as shown in Figure 2. Bu_4_NPF_6_ was used as a supporting electrolyte with a scan rate of 200 mV s^–1^. The working electrodes consisted of ITO that had been modified, while a platinum wire and a Ag/Ag electrode were used as the counter and reference electrodes, respectively. The only positive region of the voltammogram was scanned because the application of negative potential causes the cleavage of anchoring groups from the ITO surface. Since fluorine on SAM structures is an electron-withdrawing group, the oxidation potential of SAM molecules increases with the increasing number of fluorines. This phenomenon confirms the successful modification of the ITO surface with SAM molecules (Table 1.

**Table 1:** Energy Levels of HOMO for the SAMs on the Surface of the ITO Electrodes

molecules	*V*_ox_ (V)	*E*_HOMO_ (eV)
ZE-Ph	1.10	5.50
ZE-1F	1.30	5.70
ZE-2F	1.47	5.87

**Figure 3:**
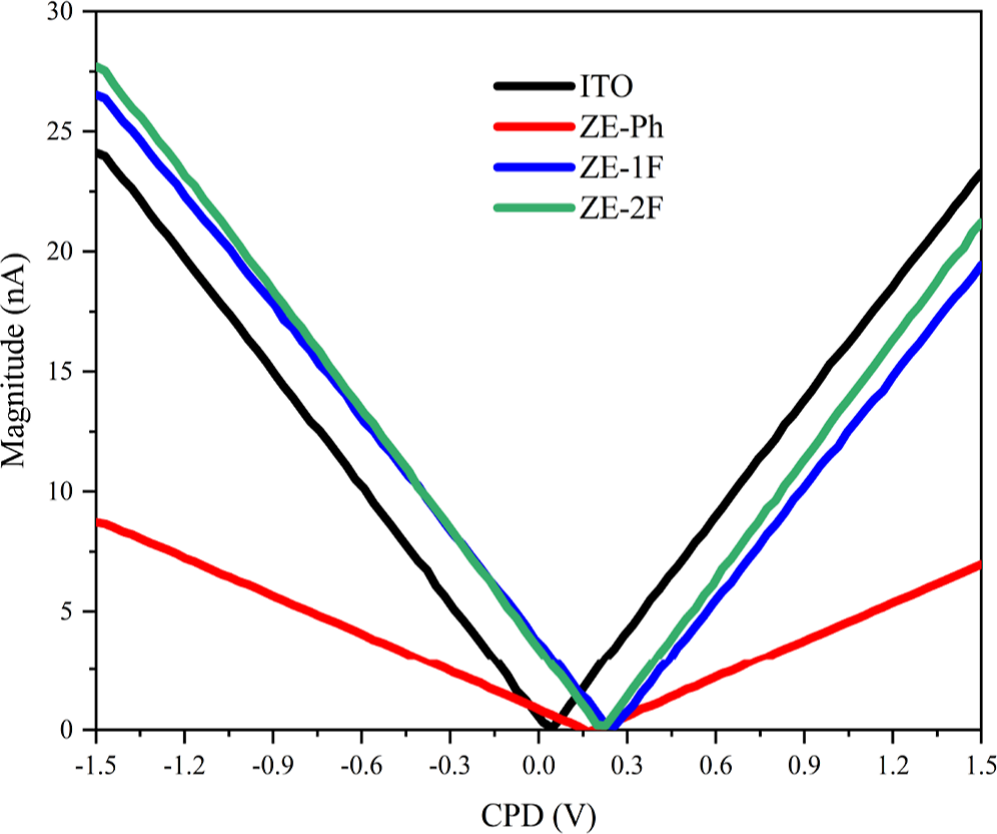
Change of CPD of bare, ZE-Ph, ZE-1F, and ZE-2F-modified ITO electrodes.

In order to determine the variations in work function resulting from the surface modification of ITO electrodes, the CPD was measured using KPFM.^36^ Average CPD values are summarized in Table 2. The CPD value between the bare ITO and Pt wire was measured as 0.0020 V, whereas it increased to 0.162, 0.223, and 0.270 V for ZE-Ph, ZE-1F, and ZE-2F after the modification of the ITO electrode as seen in Figure 3. The observed increases in CPD led to a corresponding decrease in the work function of the SAM-modified ITO electrode at the interface. The decrease in the work function of the SAM-modified ITO electrode strongly depends on the surface dipole moment. The decrease in work function values shows that the surface dipole moment direction changes toward the ITO surface.^37^ Upon the occurrence of esterification reaction between the carboxylic acid part of SAMs and the ITO surface, an accumulation of partial negative charges primarily occurs on the carboxylate part. Consequently, counterpartially positive charges are formed on the ITO surface. As a result, the effective work functions of ITO/ZE-Ph, ITO/ZE-1F, and ITO/ZE-2F are lower compared to those of the bare ITO.

**Table 2:** Average Value of Contact Potential Difference between the Surfaces and the Pt Tip^a^

anode electrode configuration	work function (eV)	CPD (V)
ITO	4.70	0.020
ITO/ZE-Ph	4.56	0.162
ITO/ZE-1F	4.50	0.223
ITO/ZE-2F	4.46	0.270

a All measurements were taken over at least 4 points.

**Figure 4:**
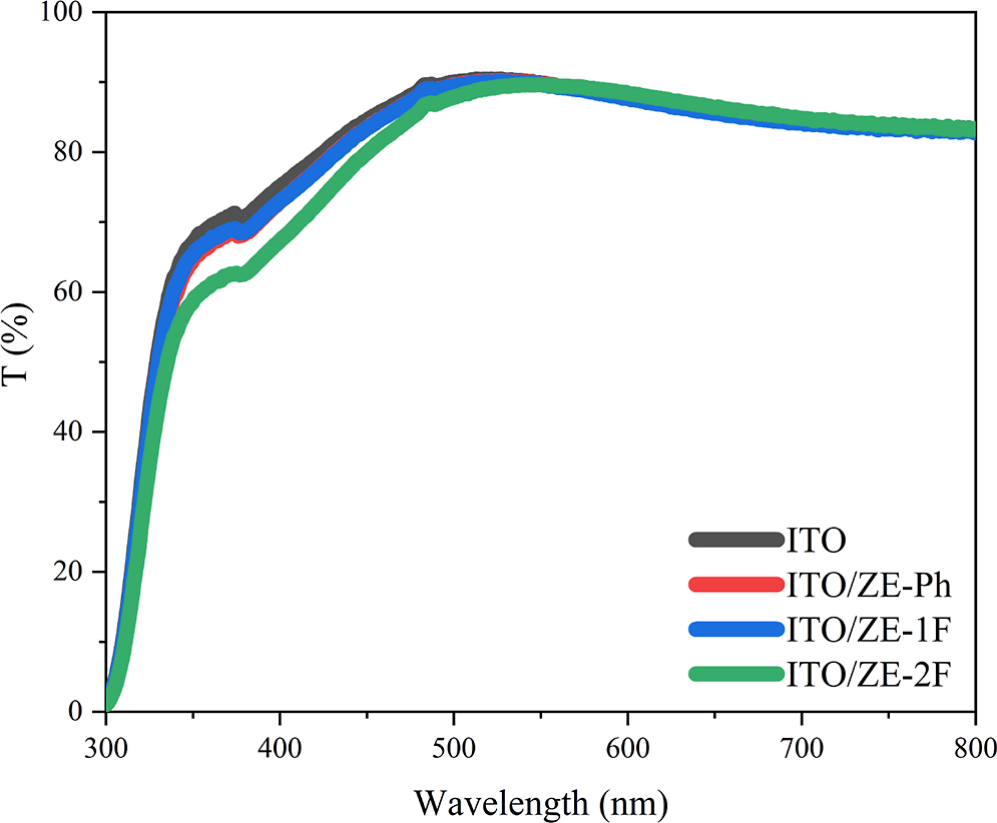
Optical transmittance spectra of the ITO and ITO/SAM films.

Transmittance measurements using UV–visible spectra were conducted to assess the impact of SAM modification on ITO substrates, as depicted in Figure 4. It was observed that the transmittance values of the ITO substrates remained nearly unchanged after the SAM treatments. This suggests that the modifications made through the ZE-Ph, ZE-1F, and ZE-2F molecules have a negligible effect on the transmittance of the ITO substrates.^38^

**Figure 5:**
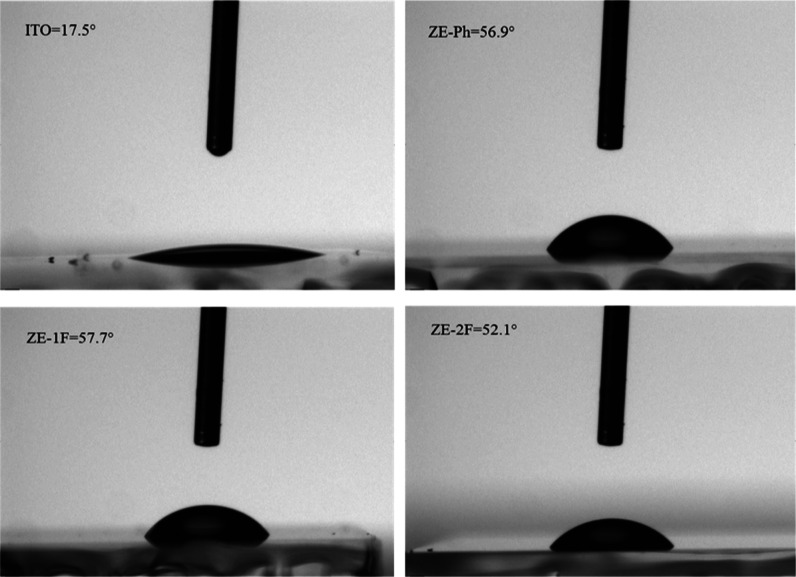
Water contact angle images for ITO, ITO/ZE-Ph, ITO/ZE-1F, and ITO/ZE-2F electrodes.

According to the literature, contact angle measurement is widely recognized as a noteworthy method for evaluating the surface characteristics of an electrode both prior to and following modification. In general, surface attachment of SAMs is expected to increase the contact angle due to the saturation of the free groups, such as OH, on the electrode surface. Figure 5 indicates the static contact angle of the electrode surface before and after modification. For each surface, 2 μL of deionized water was dropped to three different points of the electrode surfaces; subsequently, the average contact angle value was obtained. As summarized in Table 3 and depicted in Figure 4, there is a substantial increase in contact angle values after SAM coverage due to the saturation of polar groups that interact with water and lead to enhanced wettability on the surface. It is apparent from these results that the proposed SAMs are effective structures to modify ITO electrode surfaces.

**Table 3:** Contact Angle Values of the Unmodified and SAM-Modified ITO Surface

anode electrode configuration	contact angle values (deg)
ITO	17.5
ITO/ZE-Ph	56.9
ITO/ZE-1F	57.7
ITO/ZE-2F	52.1

**Figure 6:**
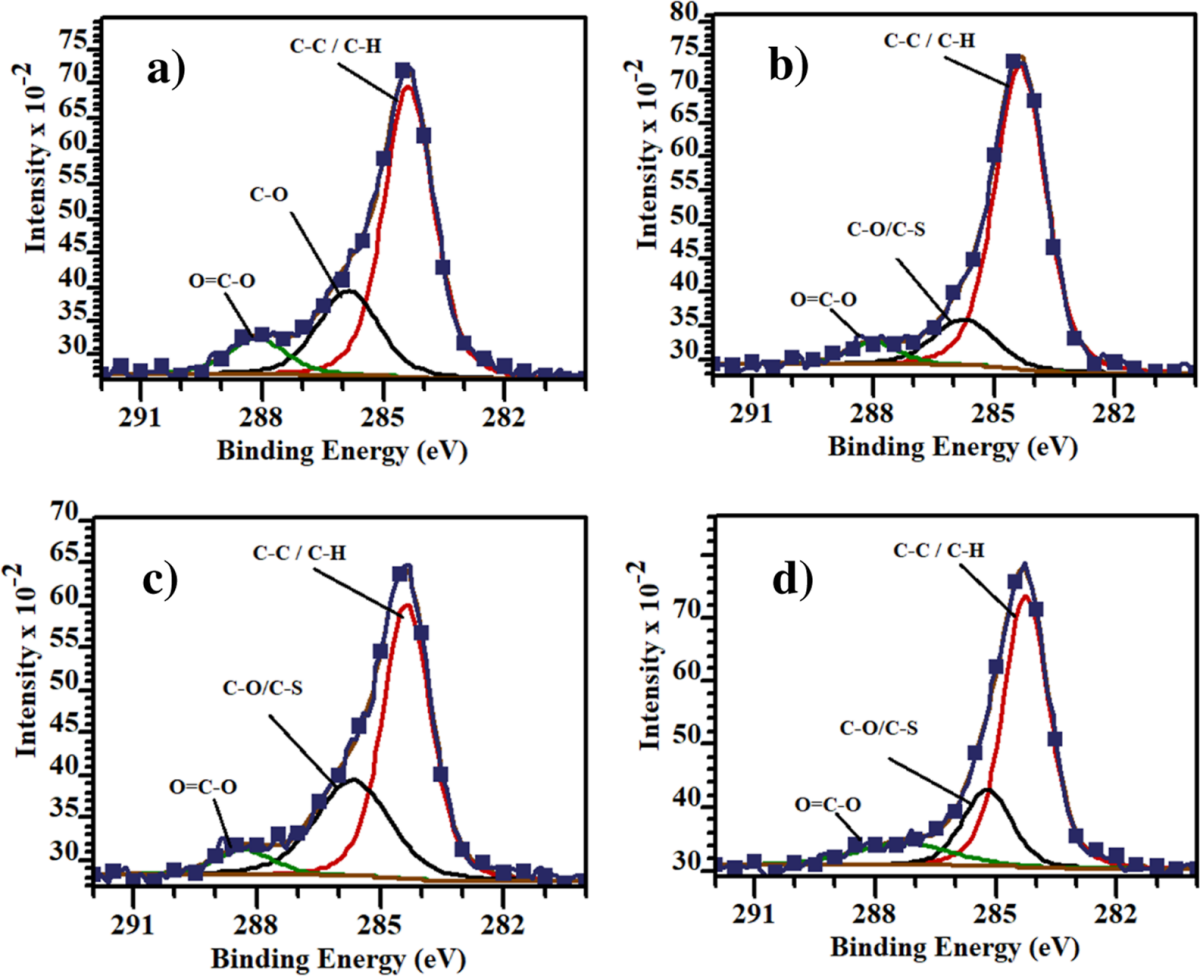
High-resolution C 1s peak spectra were obtained for the following surfaces: (a) bare ITO, (b) ZE-Ph-modified ITO, (c) ZE-1F-modified ITO, and (d) ZE-2F-modified ITO surfaces.

The X-ray photoelectron spectroscopy (XPS) technique is an effective method to determine the chemical compositions of the ITO surface modified with ZE-Ph, ZE-1F, and ZE-2F SAM molecules. High-resolution spectra of the C 1s peak obtained from the surface of ITO and ITO/SAMs are shown in Figure 6. The presence of ZE-Ph, ZE-1F, and ZE-2F molecules on the ITO surface is strongly supported by the observation of the O=C–O covalent bonding at specific binding energies of 287.99, 288.32, and 287.30 eV, respectively. The C 1s spectrum analysis revealed three distinct components for the bare ITO electrode, including C–C/C–H bonds at 284.38 eV, C–O/C–S bonds at 285.87 eV, and O=C–O bonds at 288.13 eV. For the ZE-Ph-modified ITO surface, the C 1s spectrum exhibited peaks at 284.34, 285.74, and 287.99 eV. Similarly, for the ZE-1F-modified ITO surface, the peaks appeared at 284.36, 285.66, and 288.32 eV. For the ZE-2F-modified ITO surface, the peaks appeared at 284.25, 285.21, and 287.30 eV. These findings provide strong evidence for the successful surface coverage of SAM molecules onto the ITO.^39,40^ For the ITO surface, the peak at 529.45 eV corresponds to the lattice oxide, i.e., O_2_^–^, while the peaks at 531.04 eV correspond to C=O.^40,41^ After the ITO surface was modified with ZE-Ph, ZE-1F, and ZE-2F, these peaks were located at 529.48 and 530.79 eV, 529.53 and 530.88 eV, and 529.54 and 530.72 eV, respectively. The binding energy of the S 2p peak for all 3 molecules is 168.5 eV (Figures S1–S4).^42^ Additionally, the F 1s XPS spectrum for ZE-1F and ZE-2F molecules at 689.3 eV is another indication that the molecules are attached to the surface (Figure S5).^43^ The corresponding binding energy values of the ITO, ITO/ZE-Ph, ITO/ZE-1F, and ITO/ZE-2F surfaces are summarized in Table 4.

**Table 4:** C 1s, O 1s, and S 2p Binding Energy Values for Bare ITO, ZE-Ph, ZE-1F, and ZE-2F-Modified ITO Surfaces

	O 1s (eV)		C 1s (eV)			
samples	O_2–_	C=O	C–C and C–H	C–O/C–S	O=C–O	S 2p (eV)
ITO	529.45	531.04	284.38	285.87	288.13	
ITO/ZE-Ph	529.48	530.79	284.34	285.74	287.99	168.5
ITO/ZE-1F	529.53	530.88	284.36	285.66	288.32	168.5
ITO/ZE-2F	529.54	530.72	284.25	285.21	287.30	168.5

**Figure 7:**
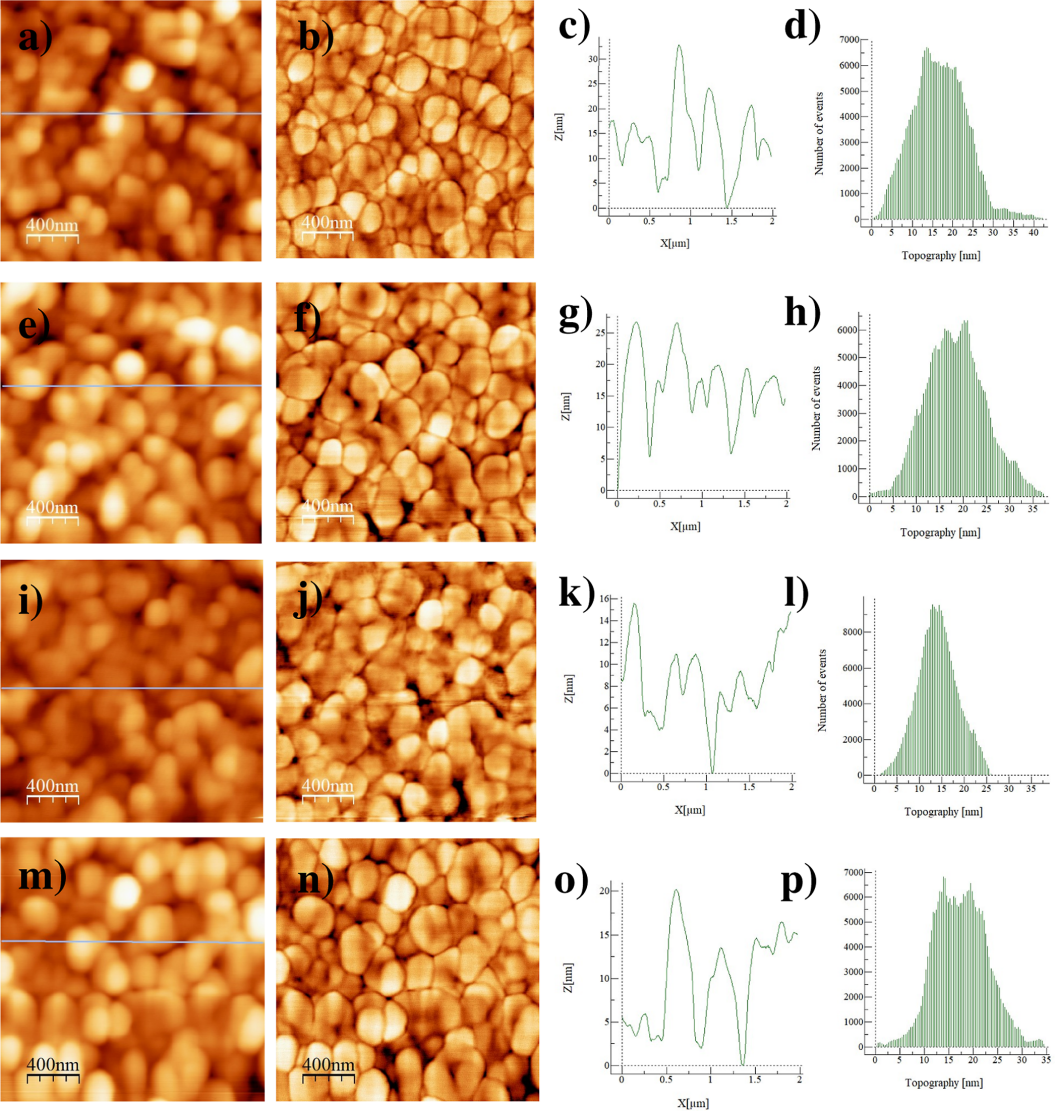
2 × 2 μm AFM images, phase images, profile, and topography of (a–d) ITO, (e–h) ITO/ZE-Ph, (i–l) ITO/ZE-1F, and (m–p) ZE-2F-modified ITO surfaces.

Atomic force microscopy (AFM) was used to measure the surface topography and root-mean-square surface roughness of SAM molecules on ITO. AFM images of bare ITO and ITO/ZE-Ph, ITO/ZE-1F, and ITO/ZE-2F-coated surfaces do not show a major difference between SAM-treated surfaces (Figure 7). While the surface roughness is 6.65 nm for bare ITO, it is 6.36 nm for the ZE-Ph-coated film, 4.47 nm for the ZE-1F-coated film, and 5.36 nm for the ZE-2F-coated film, indicating the formation of a uniform and flat molecular film. A smoother surface will provide better interface contact, thus positively affecting the device performance. We can conclude that modifying ITO with these molecules will not have a significant effect on the morphology of the upper layers.^44^

**Figure 8:**
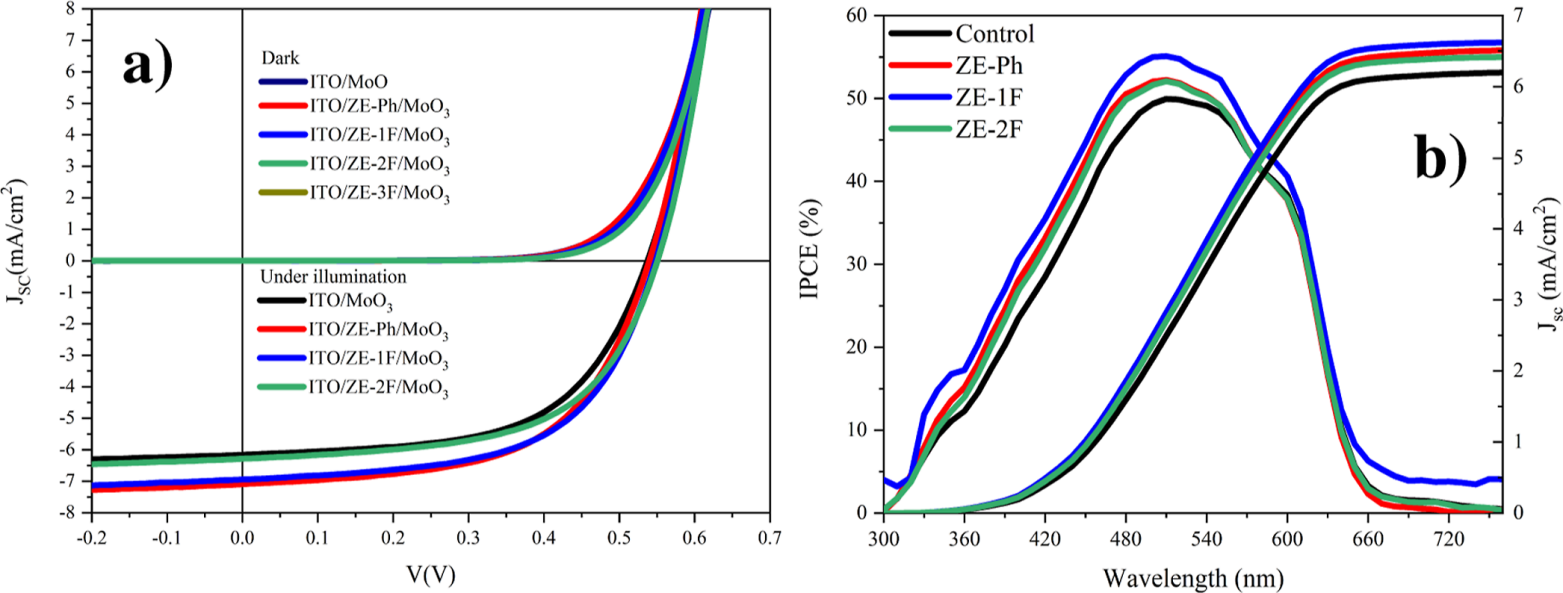
(a) Dark and light current density–voltage (*J*–*V*) curves of unmodified ITO and SAM-modified ITO devices. (b) IPCE spectra of unmodified and SAM-modified ITO-based OSCs.

For evaluating the effect of SAMs on the OSC’s performance, ITO/SAM/MoO_3_ anode buffer layer electrode configurations were used in device fabrication. Figure 8a shows the dark and light current density–voltage (*J*–*V*) plots of OSCs using both bare ITO/MoO_3_ and SAM-modified ITO/SAM/MoO_3_. Table 5 shows the photovoltaic parameters, including the average and best power conversion efficiency (PCE), obtained from *J*–*V* plots and electrical characterizations of solar cells measured under room-temperature conditions with an irradiance of 100 mW/cm^2^ and A.M. 1.5G. The ITO/ZE-1F/MoO_3_ electrode had the highest PCE value of 2.22%, while the PCE value of the standard device was found to be 1.93%. The other PCE values are 2.20 and 2.01% for the ITO/ZE-Ph and ITO/ZE-2F electrodes, respectively. The short-circuit current (*J*_sc_) values of fabricated OSCs were increased by the use of ZE-Ph and ZE-1F as given in Table 5. The highest *I*_sc_ value was obtained as 7.10 mA/cm^2^ with the ITO/ZE-Ph electrode configuration, while the *J*_sc_ value of the standard device was obtained as 6.16 mA/cm^2^.

**Table 5:** Best and Average Obtained Photovoltaic Parameters of Unmodified and SAM-Modified ITO Devices^a^

parameters		control	ZE-Ph	ZE-1F	ZE-2F
*J*_SC_ (mA/cm^2^)	best	6.16	7.10	6.94	6.28
	average	6.02	6.93	6.90	6.20
*V*_OC_ (mV)	best	540	540	550	550
	average	533	540	550	547
FF (%)	best	57.9	57.3	58.0	58.2
	average	56.4	56.6	56.7	56.4
PCE (%)	best	1.93	2.20	2.22	2.01
	average	1.85	2.12	2.14	1.90
*R*_s_ (Ω cm^2^)	best	20.7	15.0	15.2	16.9
*R*_p_ (Ω cm^2^)	best	710.0	772.2	793.0	804.5

a Data were averaged from nine individual devices.

The observed increase in the *J*_sc_ value can be primarily attributed to the improved ohmic contact between the ITO and MoO_3_ interface facilitated by the modification of SAMs. The mismatch in the Fermi level between ITO and the conduction band of MoO_3_ leads to a reduction in the *J*_sc_. However, by adding SAM molecules into the ITO/MoO_3_ interface, the effective energy barrier level encountered by hole charge carriers injected from MoO_3_ or electron charge carriers injected from ITO is reduced. The results show that the *J*_sc_ values obtained from solar cells fabricated with SAM-modified ITO electrodes show improved *J*_sc_ values compared to those of the control device. Additionally, the observed increase in the *V*_oc_ value can be attributed to the alteration in the effective work function of ITO, resulting in Fermi-level pinning at the interface between ITO and MoO_3_.^45,46^

SAM-modified OSCs have lower *R*_s_ values than those of the control device, while OSCs with SAM-modified ITO electrodes have higher *R*_p_ values than those of the control device. The OSCs with unmodified ITO, ITO/ZE-Ph, ITO/ZE-1F, and ITO/ZE-2F electrodes exhibit calculated *R*_p_ values of 710.0, 772.2, 793.0, and 804.5 Ω·cm^2^, respectively. Additionally, the calculated *R*_s_ values for these devices are 20.7, 15.0, 15.2, and 16.9 Ω·cm^2^ for unmodified ITO, ITO/ZE-Ph, ITO/ZE-1F, and ITO/ZE-2F, respectively. The fill factor value of OSCs is also improved by the use of SAMs compared with bare ITO as given in Table 5. By SAM engineering at the ITO/MoO_3_ interface: (i) the electrical contacts at the interfaces were improved, (ii) the surface roughness of the ITO surface was reduced, and (iii) the interfacial dipole was adjusted. As a result, OPV devices showed improvement in both best and average photovoltaic properties in nine devices by improving the ITO/MoO_3_ interface, resulting in a PCE gain of approximately 14%. Similar results were reported with the ITO/SAM/ZnO-modified study by Hsu et al.^34^ The results show the importance of interface modification between the ITO and MoO_3_ buffer layers to effectively improve the change barrier height in the inorganic part of OSCs and the solar cell parameter. Figure 8b shows the graphs of the IPCE for the OSCs. IPCE represents the ratio of the number of incident photons to the number of charge carriers collected in the external circuit. The IPCE value is determined by the following equation^47^
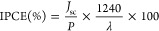
1where *J*_sc_ is the short-circuit current density (A/cm^2^), *P* is the incident power (W/cm^2^), and λ is the wavelength (nm).

From Figure 8, it can be observed that the IPCE value of the unmodified ITO device was 49.95% at a wavelength of 510 nm, which corresponds to the maximum absorption peak of P3HT. The highest IPCE value was obtained as 55.10% with the ITO/ZE-1F device, which is higher than that of ITO/ZE-Ph (52.22%), ITO/ZE-2F (52.07%), and bare ITO (49.95%). Figure 7 presents the IPCE spectra collected within the 300–750 nm range along with the corresponding integrated current density (*J*_sc_) values. The *J*_sc_ values derived from the IPCE measurements were 6.208, 6.523, 6.629, and 6.423 mA/cm^2^ for control, ZE-Ph, ZE-1F, and ZE-2F, respectively. As a result, all the SAM-modified devices exhibited higher IPCE values than those of the unmodified ITO devices, correlating well with the *J*–*V* data. Our findings provide highly convincing evidence that interface modification between the active and charge transport layers is a crucial strategy for enhancing the efficiency of solar cells by improving the *J*_sc_, *V*_oc_, FF, *R*_s_, and *R*_p_ values.

## 4 Conclusions

The focus of this study was to investigate the impact of SAM modification on the ITO/MoO_3_ interface in OSC applications. Monolayer formation was systematically used to modify the surface of the ITO anode layer by utilizing self-assembly by carboxylic acid-terminated molecules. The utilization of SAM molecules for modifying the ITO surface led to enhancements in OSC electrical parameters, including *V*_oc_, *I*_sc_, FF, and PCE. The main contributing factor to these improvements is the energy level tuning between the ITO and MoO_3_ interface. Utilizing SAM molecules as an approach to enhance the solar cell efficiency emerges as a promising method for developing organic active layers, offering both ease of application and effectiveness. By ensuring compatibility between the energy levels of SAM molecules and charge transport layers at the interface, the device efficiency is improved, leading to an increase in the electrical characterization parameters of OSCs.

## Data Availability

The data file will be made available on request.

## Supporting Information Available

XPS survey spectra and high-resolution spectra of the F 1s peak (PDF)
