# Supplementary material for: Effect of Anode Interfacial Modification by Self-Assembled Monolayers on the Organic Solar Cell Performance
Source: ACS Omega. 2024 Feb 7;9(7):7413–23. doi: 10.1021/acsomega.3c04081 (PMC10882651; doi:10.1021/acsomega.3c04081)
Supplement: Supplementary file 1 — ao3c04081_si_001.pdf [file ao3c04081_si_001.pdf]

Supporting Information

The effect of anode interfacial modification by self-assembled monolayers on the organic solar cell performance

Adem Mutlu[a*], M. Zeliha Arkan[b], Mustafa Can[c], Cem Tozlu[c*]

[a]*Solar Energy Institute, Ege University, 35100, Izmir, Turkey*

[b]*Institute of Chemistry, University of Silesia in Katowice, Szkolna 9, Katowice 40-006, Poland*

[c]*Graphene Application and Research Center, Izmir Katip Celebi University, Cigli, 35620 Izmir, Turkey*

Corresponding authors: Tel.: +90 2323115011, E-mail adresses: adem.mutlu@ege.edu.tr (A. Mutlu), Tel.: + 90 232 3293535, E-mail: cem.tozlu@ikcu.edu.tr (C. Tozlu)

This file includes:

Supplementary Figures S1-S5

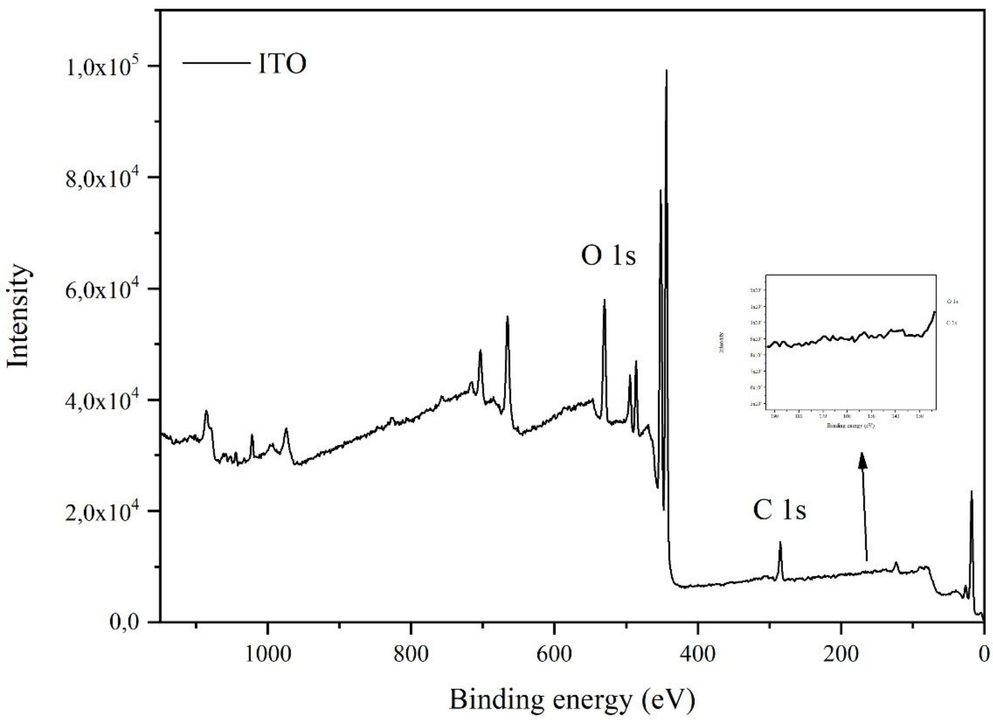

Figure S1. XPS survey spectra ITO.

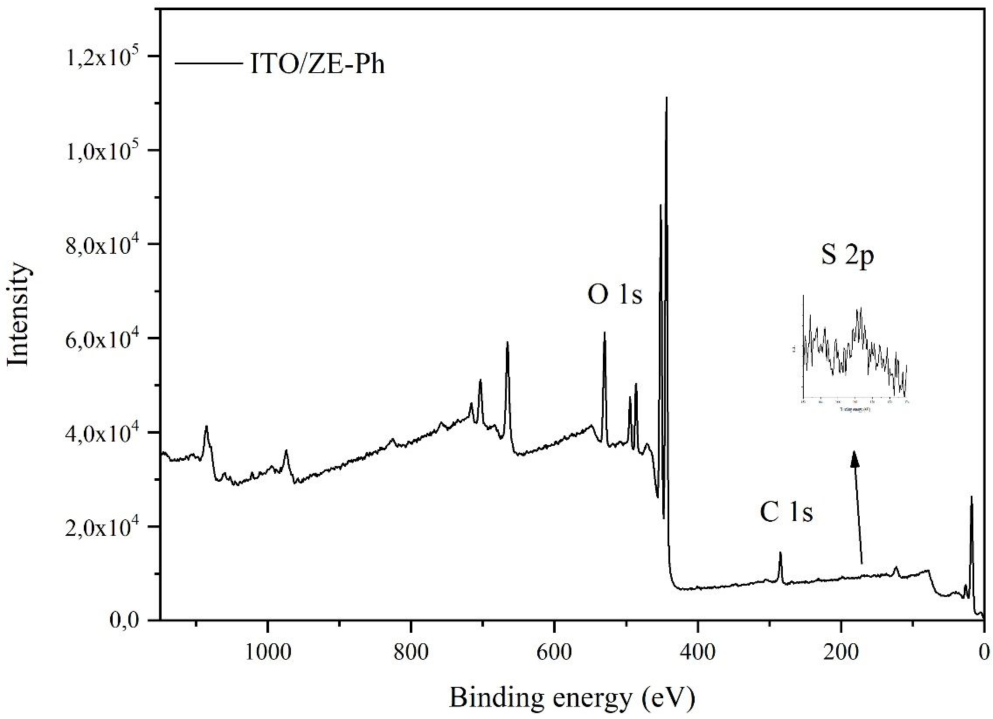

Figure S2. XPS survey spectra of ZE-Ph modified ITO.

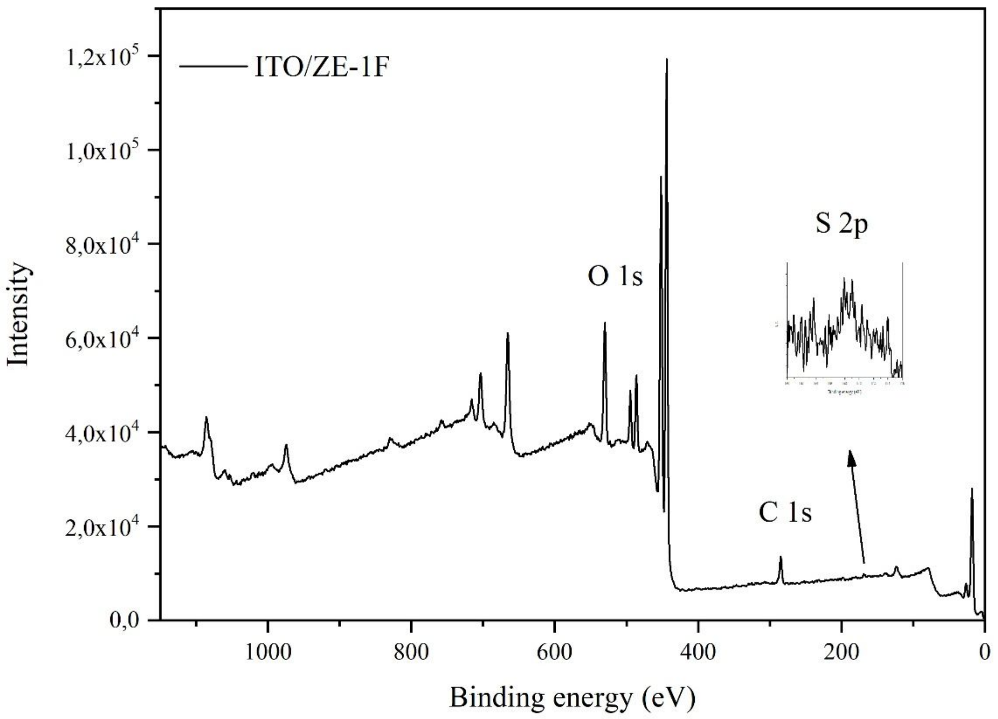

Figure S3. XPS survey spectra of ZE-1F modified ITO.

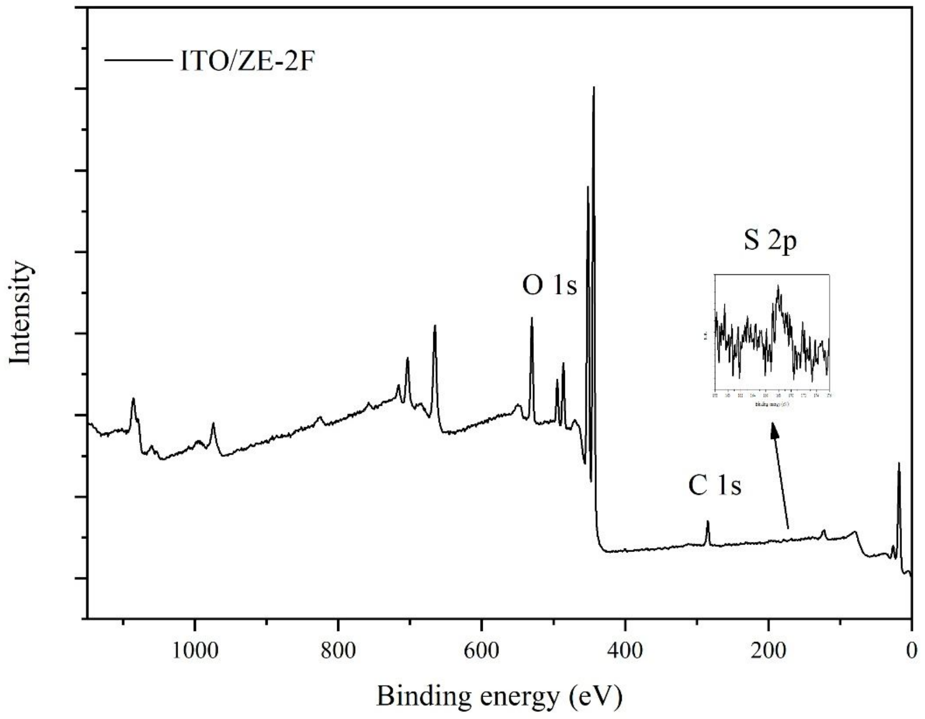

Figure S4. XPS survey spectra of ZE-2F modified ITO.

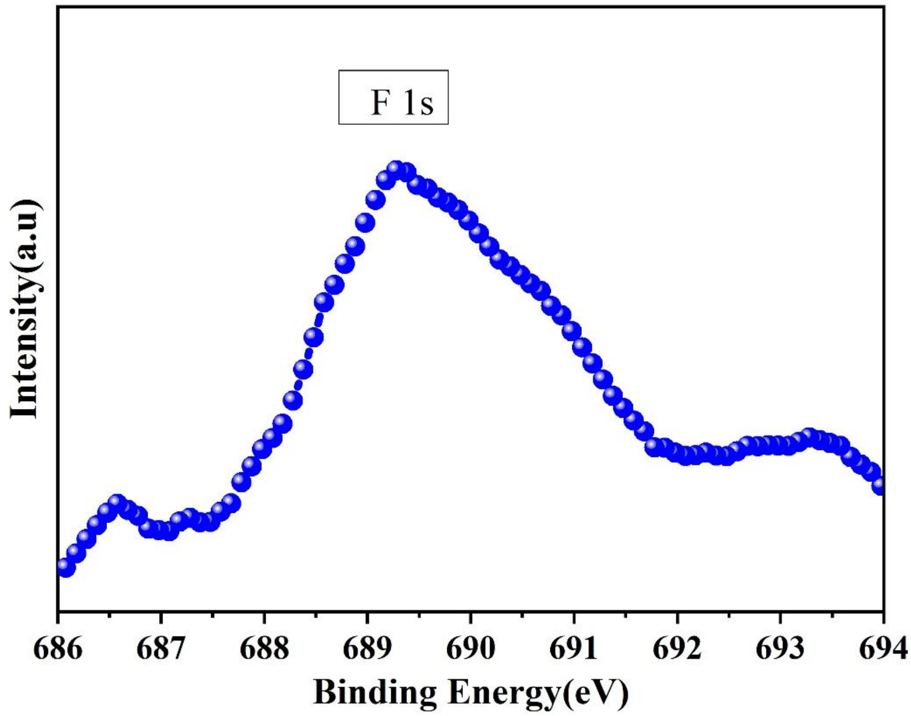

Figure S5. The high resolution spectra of F 1s peak of ZE-1F and ZE-2F modified ITO.
